# Pulmonary Delivery of Curcumin and Beclomethasone Dipropionate in a Multicomponent Nanosuspension for the Treatment of Bronchial Asthma

**DOI:** 10.3390/pharmaceutics13081300

**Published:** 2021-08-20

**Authors:** Luca Casula, Francesco Lai, Elena Pini, Donatella Valenti, Chiara Sinico, Maria Cristina Cardia, Salvatore Marceddu, Giorgia Ailuno, Anna Maria Fadda

**Affiliations:** 1 Dipartimento di Scienze della Vita e dell’Ambiente, Sezione di Scienze del Farmaco, Università degli Studi di Cagliari, 09124 Cagliari, Italy; luca.casula@unica.it (L.C.); frlai@unica.it (F.L.); valenti@unica.it (D.V.); sinico@unica.it (C.S.); cardiamr@unica.it (M.C.C.); 2 DISFARM, Sezione di Chimica Generale e Organica “A. Marchesini”, Università degli Studi di Milano, Via Venezian 21, 20133 Milano, Italy; elena.pini@unimi.it; 3 Istituto di Scienze delle Produzioni Alimentari (ISPA)-CNR, Sez. di Sassari, 07040 Baldinca, Italy; salvatore.marceddu@cnr.it; 4 Department of Pharmacy, University of Genova, 16147 Genova, Italy; ailuno@difar.unige.it

**Keywords:** curcumin, beclomethasone dipropionate, nanosuspension, asthma, pulmonary delivery, NGI, DSC, XRPD, ATR-FTIR

## Abstract

Curcumin has shown a potential extraordinary activity as an add-on ingredient in asthma treatment, due to its immunomodulatory and anti-inflammatory mechanism of action. However, its low water solubility and bioavailability lead to a poor therapeutic effect, which can be overcome by its formulation as nanocrystals. The aim of this study was to prepare a multicomponent formulation for the delivery of curcumin (CUR) and beclomethasone dipropionate (BDP) into the lungs as water-based nanosuspensions (NS). Single component formulations (CUR-NS, BDP-NS) and a multicomponent formulation (CUR+BDP-NS) were prepared through a wet ball media milling technique, using P188 as a non-toxic stabilizer. Characterization was carried out in terms of size, size distribution, zeta potential, nanocrystals morphology, and solid-state properties. Moreover, the inhalation delivery efficiency was studied with Next Generation Impactor (NGI, Apparatus E Ph. Eu). CUR-NS was optimized and showed a long-term stability and improved nanocrystals apparent solubility. The three formulations exhibited a nanocrystal mean diameter in the range of 200–240 nm and a homogenous particle size distribution. Aggregation or sedimentation phenomena were not observed in the multicomponent formulation on 90 days storage at room temperature. Finally, the nebulization tests of the three samples showed optimal aerodynamic parameters and MMAD < 5 µm.

## 1. Introduction

Bronchial asthma is a chronic inflammatory disease characterized by a complex interplay of airway inflammation and hyper-responsiveness, reversible airway obstruction, mucus hypersecretion, and pulmonary edema. The common main symptoms include cough, chest tightness, dyspnea, and wheezing [1,2]. The well-known ‘umbrella’ asthma diagnosis helps to describe the heterogeneity of the disease and to identify the involved endotypes and phenotypes [3]. In particular, the prevalent phenotypes can be classified as: early-onset allergic, late-onset eosinophilic, exercise-induced, obesity-related, and neutrophilic [4]. As regards the pathogenesis of asthma, the disease development is associated with the expression of several transcription factors and, in particular, the Nuclear Factor- κB (NF-κB) [5]. Medications for the long-term treatment of asthma can be classified into: (i) controller medications to control the symptoms and reduce exacerbations, (ii) reliever/rescue medications to provide immediate relief of breakthrough symptoms, and (iii) add-on therapies for patients with severe asthma. Treatment includes inhaled corticosteroids (CS), long-acting beta2-agonist (LABA), leukotriene receptor antagonist (LTRA), oral corticosteroids (OCS), and short-acting beta2-agonist (SABA) [6]. In recent years, an increasing interest in complementary and alternative treatments in asthma patients has been shown. Natural extracts, also known as herbal medicinal products, are the most used complementary products or health-promoting agents due to their health benefits and reduced side effects [7]. Among others, curcumin, a polyphenol extracted from the rhizome of Curcuma longa, has shown a potential therapeutic value and promising pharmacological activities in a variety of chronic diseases, including bronchial asthma [8]. Its antioxidant and anti-inflammatory activities act synergically to stop the inflammatory process. In particular, curcumin's ability to attenuate airway inflammation seems to be due to the inhibition of NF-kB in the asthmatic lung tissue, which is highly involved in the pathogenesis of the disease [9,10]. Moreover, levels of pro-inflammatory and pro-fibrotic cytokines, chemokines, and heat shock proteins were found to be reduced by the polyphenol action, whereas aquaporin expression increased, leading to the reduction of pulmonary edema [11]. However, the low aqueous solubility is limiting for its potential therapeutic applications. A possible strategy to improve the pulmonary delivery of curcumin might be its administration as nanocrystals [12]. Nanocrystals suspensions are nanoparticles of pure drug without any matrix material, suspended in an outer liquid phase, usually composed of water and/or water-miscible solvents, and stabilized using an ionic or non-ionic surfactant or polymers [13]. The drug nanocrystals average diameter is below 1 µm (typically in the range of 200–500 nm). Due to the increased particle surface area and the decreased diffusion layer thickness (compared to coarse and micronized drugs), the dissolution rate is sped up, as described by the Prandtl equation [14]. The Freundlich–Ostwald equation shows that nanocrystals are also characterized by an enhanced saturation solubility [15]. Furthermore, poorly soluble drugs for lung delivery have shown to have superior pharmacokinetics properties when formulated as nanocrystals, compared to solutions or coarse suspensions of the same drug [16,17,18,19,20,21]. Therefore, the aim of our work was to formulate a multicomponent nanocrystal suspension for the inhalation therapy, composed of beclomethasone dipropionate, a corticosteroid agent, well-known for its activity to reduce the symptoms [6,22], and curcumin as a natural complementary agent. At first, curcumin nanosuspension (CUR-NS) was prepared by a top down media milling method [23]. The multi component nanosuspension (CUR+BDP-NS) was then prepared using a beclomethasone dipropionate nanosuspension (BDP-NS) studied in our previous work [24]. Characterization of the nanosuspensions was carried out via different techniques: dynamic light scattering (DLS), scanning electron microscopy (SEM), differential scanning calorimetry (DSC), X-ray powder diffractometry (XRPD), and Attenuated Total Reflectance-Fourier Transform Infrared (ATR-FTIR) spectroscopy. Finally, nebulization tests with Next Generation Impactor (NGI, Apparatus E Ph. Eu) were carried out to study the aerodynamic properties of the obtained formulations.

## 2. Materials and Methods

### 2.1. Materials

Beclomethasone dipropionate, curcumin, Kolliphor P188 (Poloxamer 188, P188) were obtained from Sigma Aldrich (Milan, Italy). All the other products were of analytical grade.

### 2.2. Preparation of Nanosuspension

The nanosuspensions were prepared through a wet ball media milling technique, using a 2:1 (*w*/*w*) drug:stabilizer ratio. The drug was dispersed in a (0.5 and 1%, *w*/*w*) Poloxamer 188 (P188) water solution using an Ultra Turrax T25 basic (IKA, Werke, Staufen, Germany) for 6 min at 8000 rpm. This coarse suspension was divided in 1.5 mL conical microtubes containing about 0.4 g of 0.1–0.2 mm yttrium-stabilized zirconia-silica beads (Silibeads^®^ Typ ZY Sigmund Lindner, Warmensteinach, Germany). For the CUR-NS, the microtubes were oscillated at 3000 rpm for 70 min using a beads-milling cell disruptor equipment (Disruptor Genie^®^, Scientific Industries, Bohemia, NY, USA). The obtained nanosuspensions of each microtube were gathered and then separated from the milling beads by sieving. As concerns the BDP-NS, the formulation was prepared as previously reported [24]. The procedure was the same as for the CUR-NS, and the oscillation time of the microtubes at 3000 rpm was 150 min. The formulations had a final concentration of 1% (*w*/*w*) active compound (CUR or BDP) and 0.5% (*w*/*w*) P188.

### 2.3. Particle Size Analysis

The average diameter and polydispersity index (PDI, as a measure of the size distribution width) of the samples were determined by Dynamic Light Scattering (DLS) using a Zetasizer nano (Malvern Instrument, Worcestershire, UK). Samples were backscattered by a helium–neon laser (633 nm) at an angle of 173° and a constant temperature of 25 °C. Zeta potential was estimated using the Zetasizer nano by means of the M3-PALS (Phase Analysis Light Scattering) technique. Just before the analysis, nanosuspensions were diluted with distilled water. Furthermore, a medium-term stability study of the CUR nanosuspension stored at room temperature was performed by average monitoring size, polydispersity index, and zeta potential for 90 days. All the measurements were made in triplicate.

### 2.4. Scanning Electron Microscopy

In order to investigate the (nano)crystals morphology, CUR raw powder and CUR-NS were analyzed through a Zeiss ESEM EVO LS 10 (Germany) environmental scanning electron microscope (SEM), operating at 20 KV in high vacuum modality with a secondary electron detector (SEI). For the CUR raw powder, the sample was mounted on an aluminum stub with carbon adhesive discs and coated with gold in an Agar Automatic Sputter Coater B7341. Regarding the CUR-NS, a drop of the sample was first placed on a glass slide and air-dried, and then mounted on the stub following the procedure stated above.

### 2.5. Solubility Studies

CUR apparent solubility in water was measured for the CUR bulk powder, CUR-NS and the CUR/P188 physical mixture with the same drug:surfactant ratio. The formulations (n = 3) were kept under constant stirring for 72 h at 37 °C. Samples were withdrawn and centrifuged at 21,380× *g* for 60 min. The supernatant was centrifuged again at 21,380× *g* for 60 min. Then, a known amount of the clear supernatant was withdrawn and diluted with methanol for the HPLC analysis.

### 2.6. Solid State Characterization

CUR, BDP, P188, physical mixtures of CUR:P188 and BDP:P188 in amounts equivalent to the ratios present in the formulations, and the 2 single-component formulations (CUR-NS and BDP-NS) were investigated by using different technologies such as DSC, XRPD, and ATR-FTIR spectroscopy.

In order to obtain the powder samples, CUR-NS and BDP-NS were frozen at −80 °C and then freeze dried for 24 h at −86 °C and 0 mm Torr, using an FDU-8606 Freeze Dryer (Operon Co., Yangchon-eup, Korea).

DSC analysis (Perkin Elmer DSC 6 Waltham, MA, USA) was used to characterize the thermal behavior of the different components used for the formulations. Samples were hermetically sealed in an aluminum pan and heated at a speed of 10 °C/min in the range between 30 and 220 °C. The inert atmosphere was maintained by purging nitrogen at a flow rate of 10 mL/min. A control empty pan subjected to the same heating conditions was used as a reference.

ATR-FT-IR spectra were acquired with a Perkin Elmer Spectrum One FT-IR (Perkin Elmer, Waltham, MA, USA), equipped with a Perkin Elmer Universal ATR sampling accessory consisting of a diamond crystal. Analyses were performed in a spectral region between 4000 and 650 cm^−1^ and analyzed by transmittance technique with 32 scansions and 4 cm^−1^ resolutions.

XRPD patterns were collected with a Rigaku MiniFlex diffractometer (Rigaku, Neu-Isenburg, Germany), operating at 30 kV and at 15 mA, with Cu Kα radiation (1.54056 Å) in the range from 3 to 60 2θ, in steps of 0.02, using a scan step time of 2.00 s. The results were then obtained as peak height (intensity) versus 2θ.

### 2.7. Preparation of Nanosuspension

CUR-NS and BDP-NS were prepared as described above (2.2). The multicomponent nanosuspension (CUR+BDP-NS) was prepared right before the nebulization test by mixing equal parts of CUR-NS and BDP-NS. The formulation was adequately vortexed and then visually inspected to check the absence of large, precipitated aggregates or phase separation. Finally, particle size analysis was carried out by DLS.

### 2.8. Nebulization and Aerodynamic Behaviour of Nanosuspensions

CUR-NS, BDP-NS, and CUR+BDP-NS were nebulized using a Pari SX^®^ air jet nebulizer attached to a Pari TurboBoy^®^ compressor (Pari GmnH, Starnberg, Germany) and connected to the Next Generation Impactor (NGI, Apparatus E, Eur. Ph 10th ed., Copley Scientific Ltd., Nottingham, UK). All the parts of the NGI were washed in methanol and allowed to dry. The collection plates were not sprayed with silicone fluid in accordance with the European Pharmaceutical Aerosol Group (EPAG) recommendations [25]. Pre-separator, which was mostly indicated for dry powder inhalers to separate very large particles and avoid blockage of NGI stages, was not used during this study.

The formulation (2 mL) was placed in the nebulizer and aerosolized to dryness directly into the throat of the NGI, using a flow rate of 15 L/min [26]. At the end of the experiment, the drug amount deposited in each stage of the impactor and the residual (undelivered) was collected, using methanol, in a glass vial, properly diluted, and analyzed by HPLC. The following nebulization parameters were evaluated: (1) the emitted dose (ED%), calculated as the percentage of drug recovered in the NGI versus the amount of drug placed in the nebulizer; (2) the fine particle dose (FPD), which represents the amount of drug contained in droplets of size less than 5 μm; and (3) the fine particle fraction (FPF%), calculated as a percentage of FPD versus the amount of drug recovered in the NGI.

The cumulative amount of drug-containing droplets with a diameter lower than the stated size of each stage was plotted as a percentage of the recovered drug versus the cut-off diameter, not including the mass deposited in the induction port due to the unavailability of a precise upper size limit for particles deposited in this section [27]. Finally, the mass median aerodynamic diameter (MMAD) of the particles was extrapolated from the graph according to the Eur. Ph. 10th ed., and the geometric standard deviation (GSD) value was calculated.

### 2.9. HPLC Analysis

Quantitative determination of BDP and CUR was performed by HPLC using a liquid chromatograph Alliance 2690 (Waters Corp, Milford, MA, USA) equipped with a photodiode array detector and a computer integrating apparatus (Empower 3). Analyses were performed with a Sunfire C18 column (3.5 µm, 4.6 mm × 150 mm, Waters). The mobile phase was a mixture of acetonitrile, water, and acetic acid (95:4.84:0.16 *v*/*v*), delivered at a flow rate of 0.5 mL/min. Samples (10 µL) were injected using an auto sampler. CUR was revealed at 421 nm, whereas BDP was at 240 nm. The stock standard solutions of CUR and BDP were prepared by dissolving the drug in methanol and stored at 4 °C. A standard calibration curve (peak area of CUR/BDP vs. known drug concentration) was built up by using standard solutions prepared by dilution of the stock standard solution with the mobile phase. Calibration graphs were plotted according to the linear regression analysis, which gave a correlation coefficient value (R^2^) of 0.999. The described HPLC method was also used to quantify the drug in the obtained nanosuspensions and to evaluate the presence of any degradation products. The limit of quantification was 1 ng/µL for CUR and 0.5 ng/µL for BDP, while the limit of detection was 0.2 ng/µL for both compounds. Sample preparation and analyses were performed at room temperature. 

### 2.10. Statistical Analysis of Data

Results were expressed as the mean ± SD. Multiple comparisons of means (one-way ANOVA) were used to substantiate statistical differences between groups, while Student’s *t*-test was used to compare two samples. Data analysis was carried out with the software package XLStatistic for Microsoft Excel. Significance was tested at 0.05 level of probability (*p*).

## 3. Results and Discussion

### 3.1. Preparation and Characterization of Nanosuspension

**Figure 1:**
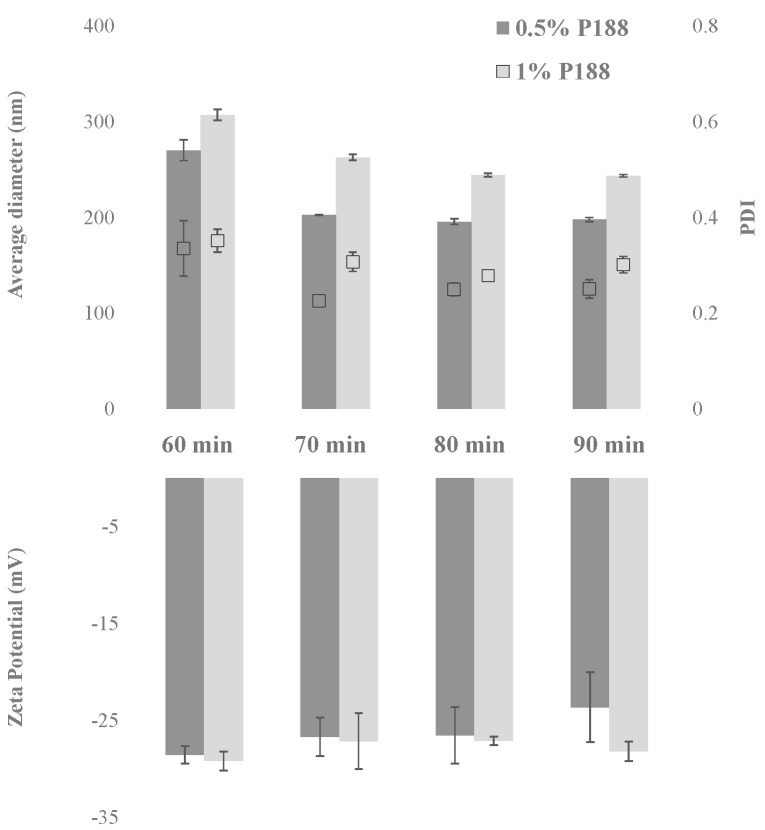
Average diameter (nm), PDI and zeta potential (mV) as a function of milling time (minutes) for the formulation with 1% (*w*/*w*) CUR and 0.5% (*w*/*w*) P188, and with 1% (*w*/*w*) CUR and 1% (*w*/*w*) P188. (n = 3; mean ± SD).

A preliminary study was carried out to optimize the protocol for the CUR-NS preparation through the wet ball media milling technique. Two parameters were investigated, namely the stabilizer concentration and the milling time. CUR concentration was fixed at 1% (*w*/*w*), whereas two concentrations of the stabilizer P188 were studied: 0.5 and 1% (*w*/*w*). The two formulations were milled for 60, 70, 80, and 90 min. Average diameter, PDI, and zeta potential as a function of the milling time are shown in Figure 1. 

Both formulations showed a significant decrease in the nanocrystal average diameter by increasing the milling time from 60 to 70 min. As highlighted in Figure 1, also, the PDI value improved for formulation with 0.5% P188, decreasing from 0.34 to 0.23. On the other hand, by increasing the milling time from 70 to 90 min, the variation of the nanocrystal dimensional properties was less pronounced. It is worth to note that after all the milling protocols, the zeta potential values were maintained at approximately −30 mV, representative of promising formulation stability. However, as can be seen in the Figure, when the 1:1 drug:surfactant ratio (*w*/*w*) was used, the PDI never decreased to values lower than approximately 0.30, even after 90 min of milling. Therefore, the formulation containing 1% CUR and 0.5% P188 obtained after 70 min of milling (CUR-NS) was selected for further studies.

CUR solubility studies were performed in water at 37 °C to evaluate the properties of nanocrystal CUR-NS in comparison with CUR raw powder and the physical mixture. The raw drug powder showed an apparent solubility of 0.97 ± 0.1 µg/mL, which increased 38-fold in the physical mixture with the P188 (38.10 ± 1.2 µg/mL). After the nanosizing process, the CUR nanocrystal apparent solubility increased further (53.08 ± 1.7 µg/mL). Consequently, the preparation of nanocrystals stabilized by P188 allowed us to improve CUR apparent solubility by approximately 54-fold in comparison with the raw material, in accordance with the Freundlich–Ostwald equation [15].

These results suggest that drugs formulated as nanocrystals would improve therapeutic efficacy. However, it is worth highlighting that, depending on the drug characteristic, increased biological medium concentration can also lead to more toxic effects. As widely reported in the literature, very challenging safety issues need to be addressed [28,29]. Indeed, many factors influence nanosuspension safety in drug delivery, such as particle surface area, formulation, external environment, and temperature. Moreover, most nanosuspension drug delivery studies were carried out in animal models instead of in humans, therefore, full knowledge of them is still limited [30].

**Figure 2:**
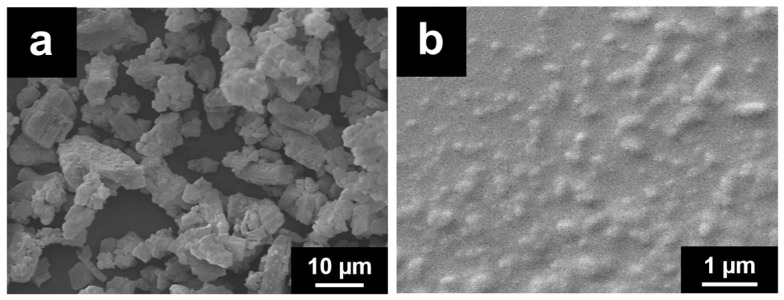
ESEM micrographs of CUR raw powder (**a**) and CUR-NS (**b**).

The evaluation of the morphological changes of CUR crystals after the milling process was carried out by ESEM (Figure 2).

As it can be seen in the ESEM micrographs, the milling process modified both the shape and size of the CUR crystals. The considerable amount of energy required to reduce the nanocrystal size below one micron is provided during the milling process by the collision of the drug crystals and the milling beads and of the drug crystals themselves, that generate high shear forces. Before the milling, Figure 2a, the raw drug material appears to have large crystals with irregular elongated shape while, after the milling with the stabilizer, as shown in Figure 2b, CUR nanocrystals show a regular and rounded shape, with a homogenous particle size distribution, in accordance with DLS analysis. 

**Figure 3:**
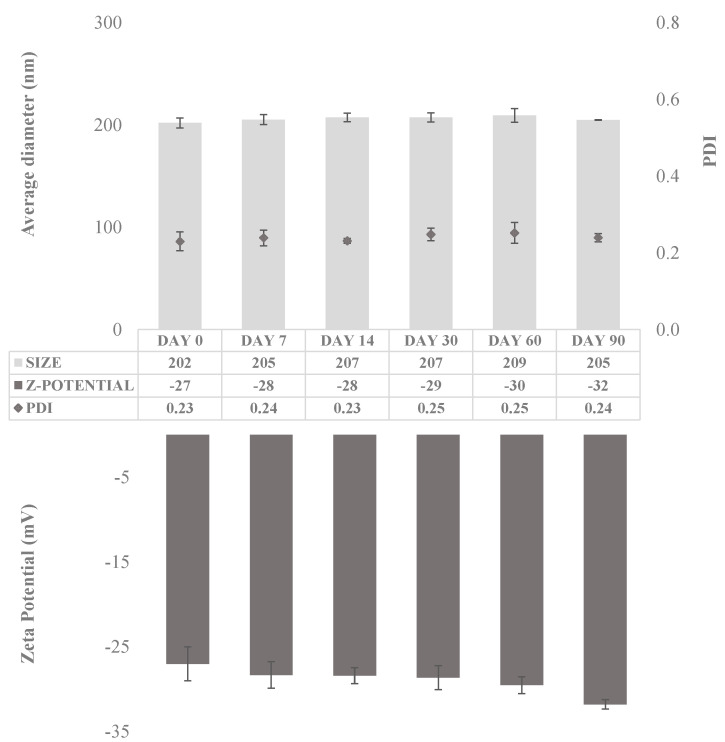
Average diameter (nm), polydispersity index (PDI), and zeta potential (mV) of CUR-NS over 90 days of storage at room temperature. (n = 5; mean ± SD).

The stability of the obtained CUR-NS was evaluated by monitoring size distribution and zeta-potential over a period of 90 days at room temperature (Figure 3).

The size distribution study revealed long-term stability of the CUR-NS. Indeed, the mean diameter did not vary appreciably during the 90 days on storage, showing an average diameter of 202 nm on day 1 and of 205 nm on day 90. Furthermore, the PDI was almost constant and below 0.25, confirming the stability of the formulation since the retention of the homogeneous size distribution on storage [31]. Moreover, the zeta potential value was almost constant during the stability test (approximately −30 mV). Finally, HPLC analysis revealed no decomposition and the absence of degradation products in the obtained chromatograms.

The final multicomponent nanosuspension (CUR+BDP-NS) was obtained by mixing CUR-NS with BDP-NS, which was prepared according to the previously reported procedure [21] with a 1% (*w*/*w*) BDP concentration and 0.5% (*w*/*w*) P188. BDP nanocrystals exhibited a mean diameter of approximately 240 nm, with a low PDI (0.24) indicating a well-dispersed colloidal dispersion.

Solid state characterization of CUR-NS, BDP-NS, and their components as raw material and the physical mixture was carried out by DSC, ATR-FTIR, and XRPD.

### 3.2. DSC Analysis

To evaluate the possible interactions between the active ingredients and the stabilizer in the preparation, thermal analysis was performed; results are expressed as onset temperature. CUR thermogram (Figures S1–S3) revealed the presence of an endothermic peak at 165.05 °C, while P188 at 53.71 °C, which implies that both are in the crystalline state. In the physical mixture, both the sharp endothermic peak of the stabilizer and the broad CUR peak showed less intensity and a shift towards lower temperatures (47.96 °C and 147.35 °C, respectively) compared to the component melting points, suggesting a molecular dispersion of CUR in P188. This trend became even more evident in the optimized formulation, thus, suggesting that the CUR existed in a less crystalline state.

As it concerns BDP-NS and its components, the BDP thermal behavior (Figures S4–S6) revealed an endothermic peak at 212.09 °C followed by an exothermic event, thus, indicating that the recrystallized BDP undergoes a melting process followed by chemical degradation. Furthermore, in the BDP-NS thermogram, an endothermic event between 60 and 90 °C is visible, suggesting the formation of hydrate BDP, as reported in the literature [32,33].

The melting peak of P188 was at an onset temperature of 53.71 °C. Physical mixture and nanosuspension thermograms showed some similarities, in fact, the melting peaks were all present but drifted, and with a sharp decrease in the BDP peak intensity, implying that no amorphous forms were produced during the preparation process.

### 3.3. ATR-FTIR Analysis

ATR spectroscopy was carried out to further elucidate the interactions between the active compounds and P188 in the solid state. These interactions were detected by any changes in the position or disappearance of a characteristic vibration or stretching region of the compounds. 

**Figure 4:**
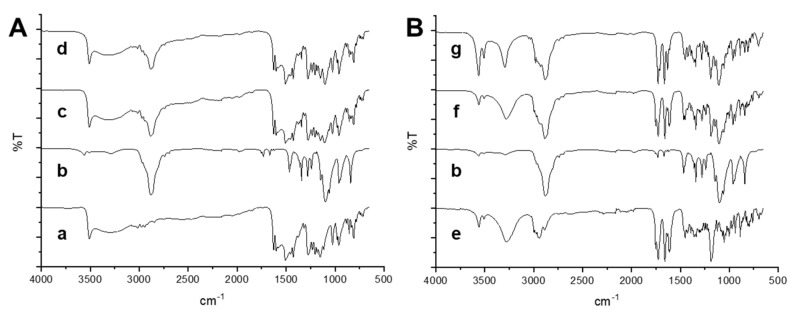
ATR-FTIR analysis of (**A**) CUR formulation and its components: raw powder (a), P188 (b). physical mixture of CUR+P188 (c), CUR-NS (d); and (**B**) BDP formulation and its components: raw powder (e), physical mixture of BDP+P188 (f), and BDP-NS (g).

The ATR spectrum of CUR (Figure 4A) exhibited a sharp peak at 3509 and a broad one at 3326 cm^−1^ attributed to phenolic OH stretching. Furthermore, it can be observed a peak at 1626 cm^−1^ owing to the carbonyl in CUR, consistent with the formation of a keto-enol tautomer, at 1602 and 1510 cm^−1^ the bands of the strong vibrations of C==C and C=O stretching, while at 1274 cm^−1^ the C–O peak of enol. At 1027 cm^−1^, the C–O–C peak was visible, while at 962 cm^−1^ and 810 cm^−1^, the trans-C-H vibration of the unsaturated chain and the C–H vibration of the aromatic ring, respectively, were clearly shown. Finally, the characteristic absorption peaks of P188 around 3600, 2881, and 1099 cm^−1^ were attributed to O–H, C–H, and C–O–C stretching vibrations. The spectrum of the physical mixture was the combination of CUR and P188. These results clearly demonstrated that no interactions occurred between the physically mixed CUR and P188. The spectra of CUR-NS exhibited the same peak position of raw CUR demonstrating that the addition of the stabilizer and physical process would not affect its molecular structure.

The ATR spectrum of BDP (Figure 4B) showed the O-H free and associated vibrations at 3559 and 3280 cm^−1^, the ester carbonyl stretching at 1753, the conjugated and non-conjugated C=O stretching bands at 1727 and 1658 cm^−1^, respectively. The C=C stretching was at 1615 and 1608 cm^−1^, and the C-O bands at 1186 cm^−1^. The characteristic absorption peaks of P188 at 3500, 2881, and 1099 cm^−1^ were attributed to O–H, C–H, and C–O–C stretching vibrations, respectively. In the physical mixture spectrum, bands of both raw materials were visible, no absence of any functional peaks or addition of new peaks, thus, revealing that there was no significant chemical interaction between the drug and P188. In the nanosuspension spectrum, as reported in our previous article [21], the COO peak, the C=O and C=C stretchings at 1711, 1663, and 1631, respectively, disappeared while a peak at 1712 cm^−1^ appeared, suggesting that the BDP carbonyl group was involved in a hydrogen bond with water. The presence of water was also confirmed by the increase of peak intensities at 3562 and 3508 cm^−1^, confirming the presence of BDP as a monohydrate.

### 3.4. XRPD Analysis

The crystalline state of the active ingredients in nanosuspensions was estimated by a XRPD study.

**Figure 5:**
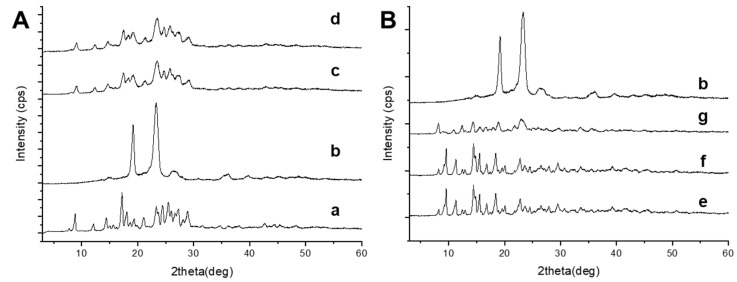
XRPD analysis of (**A**) CUR formulation and its components: raw powder (a), P188 (b). physical mixture of CUR+P188 (c), CUR-NS (d); and (**B**) BDP formulation and its components: raw powder (e), physical mixture of BDP+P188 (f) and BDP-NS (g).

The diffraction patterns of CUR (Figure 5A) showed intense sharp peaks at 8.8, 12.1, 14.4, 17.2, 18.08, 19.36, 21.08, 21.66, 23.32, 24.46, 25.48, 27.28, and 28.10 2ϑ (deg), while P188 at 19.32 and 23.48 2ϑ (deg) implying the crystalline structure of both raw materials. The physical mixture and the nanosupension profiles were very similar; reflection peaks of the raw materials were still present, indicating that CUR partially retained its crystallinity in the formulation. 

To confirm the crystalline nature of BDP nanosuspension, X-ray diffraction analysis were performed (Figure 5B). BDP and P188 have crystalline profiles. The XRPD analysis of BDP showed a pattern with sharp and intense peaks at 9.54, 11.28, 14.44, and 20.06 2θ (deg) values, P188 at 19.32 and 23.48 2ϑ (deg). The physical mixture pattern indicated that the crystalline structure remained unchanged; the characteristic peaks of the drug were still present, even if their intensities were attenuated due to the lower drug content. The optimized NS retained the crystalline profile, but the increased intensity of the peaks at 8.5° and 12 2ϑ (deg) suggested the presence of BDP monohydrate, thus confirming ATR and DSC results. 

### 3.5. Preparation of the Multicomponent Nanosuspension

After the optimization and characterization of the two single-component nanosuspensions, CUR+BDP-NS was prepared by mixing equal amounts of CUR-NS and BDP-NS right before the nebulization test. The composition of the obtained formulation is indicated in Table 1. A preliminary visual inspection revealed the absence of macroscopic precipitated aggregates or phase separation. This information was also confirmed by DLS analysis. Indeed, the nanocrystals average diameter of the CUR+BDP-NS (221 nm) did not differ appreciably from CUR-NS (202 nm) and BDP-NS (241 nm) Furthermore, the PDI maintained a value of approximately 0.25.

### 3.6. Nebulization Test

To evaluate the drug deposition and determine the aerodynamic parameters, samples (CUR-NS, BDP-NS, CUR+BDP-NS) were nebulized using the PariSX^®^ air jet nebulizer connected to the NGI. It is well known that nebulizers might generate aerosol particles with different aerodynamic diameters. In particular, only those characterized by a MMAD value in the range 5–0.5 µm were believed to deposit on the lungs [34]. Operating with a flow rate of 15 L/min, the overall range of the impactor was 0.98–14.1 µm. Notably, four stages have cut sizes in the range of 0.5–5.0 µm aerodynamic diameter, and a fifth stage only slightly larger than the upper limit [26]. Nebulization time to dryness, which is the time required to complete cessation of aerosol formation, was shown to be 10 min.

**Figure 6:**
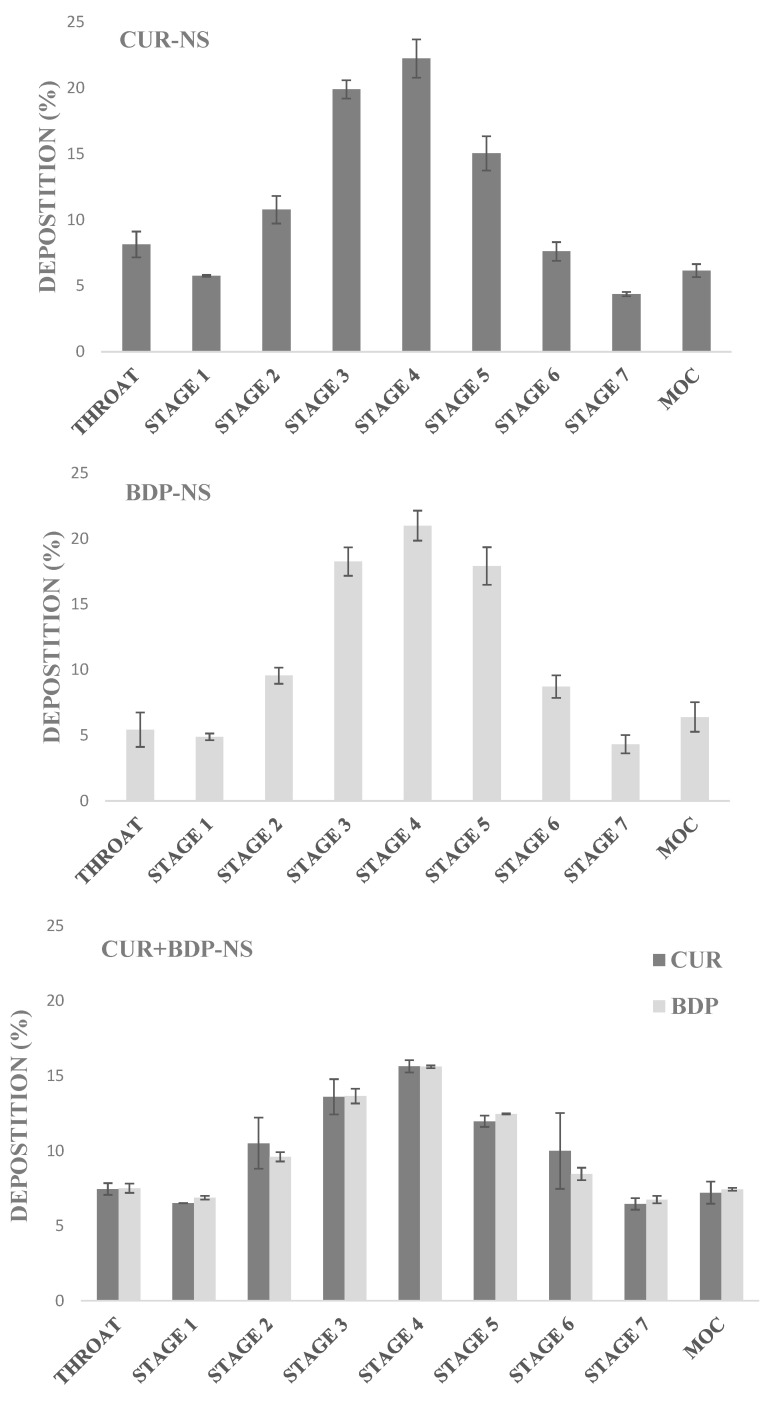
Deposition of CUR and BDP in the different stages of the NGI after nebulization with a flow rate of 15 L/min, for the single-component formulations (CUR-NS and BDP-NS), and the multicomponent formulation (CUR+BDP-NS). (n = 3; mean ± SD).

The percentage of drug deposited in each stage of the impactor was very similar for all the formulations, as shown in Figure 6.

Approximately 5–7% of the generated aerosol particles tended to deposit on the induction port (throat), thus showing their inability to reach the deeper stages. However, the majority of the drug was found to be in the intermediate/middle stages (3–5). Interestingly, approximately 6–7% of the droplets are able to reach the MOC stage, showing an aerodynamic diameter < 0.98 µm, and thus the ability to hypothetically deposit on the alveolar region of the lungs. To better evaluate the nanosuspension behavior during the nebulization process, the aerodynamic parameters were analyzed for each formulation (Table 2). In the case of CUR+BDP-NS, values were calculated separately for each active ingredient.

As can be seen, the emitted dose (ED) for CUR-NS and BDP-NS reached a value of 57% and 65.5%, respectively. It is interesting to highlight that this value increases more than 80% in the case of the multicomponent formulation, thus, demonstrating that more than 80% of the formulation loaded in the nebulizer may be properly delivered to the patient. Results showed that the mean FPF% value for the CUR in the multicomponent nanosuspension was higher than that in the CUR-NS while the opposite was for the BDP. However, statistical analysis revealed that these differences were not significant (*p* > 0.05).

Finally, all the nebulized formulations showed a MMAD < 5 µm, a mandatory condition for the droplets to be able to reach the deeper parts of the respiratory system and, therefore, to carry out their therapeutic action at the site of inflammation. 

## 4. Conclusions

In this study, a CUR nanosuspension was optimized and characterized. The resulting nanocrystals were small in size and homogeneously dispersed, showing an increased solubility compared to the bulk drug. Furthermore, the BDP-NS was successfully prepared as reported previously [24] and used for the preparation of the multicomponent nanosuspension containing CUR and BDP nanocrystals. The obtained formulation showed a narrow distribution and the absence of aggregation phenomena. In vitro nebulization tests were carried out and highlighted that all prepared formulations, especially CUR+BDP-NS, had high values of ED% and MMAD < 5 µm.

In conclusion, the obtained multicomponent nanosuspension has shown optimal dimensional properties and aerodynamic parameters, suggesting a correct and efficient delivery of the formulation in the deeper lung regions.

Owing to the improved solubility of the active ingredients formulated as nanocrystals, our formulation represents a promising lung delivery system, which can improve the course of asthmatic inflammation.

## Figures and Tables

**Table 1:** Composition of the two single-component (CUR-NS, BDP-NS) and the multicomponent formulation (CUR+BDP-NS) and their dimensional properties expressed as average diameter (nm) and polydispersity index (PDI). (n = 3; mean ± SD).

Formulations	Composition			Dimensional Analysis	
	Curcumin (% *w*/*w*)	Beclomethasone Dipropionate (% *w*/*w*)	P188 (% *w*/*w*)	Average Diameter (nm)	PDI
CUR-NS	1	-	0.5	202 ± 5	0.23 ± 0.02
BDP-NS	-	1	0.5	241 ± 2	0.24 ± 0.01
CUR+BDP-NS	0.5	0.5	0.5	221 ± 7	0.25 ± 0.02

**Table 2:** Aerodynamic parameters of the three tested formulations: emitted dose (ED), fine particle dose (FPD), fine particle fraction (FPF), mass median aerodynamic diameter (MMAD), and geometric standard deviation (GSD). (n = 3; mean ± SD). ^§^ Data are not statistically different (*p* > 0.05).

Aerodynamic Parameters	CUR+BDP-NS			
	CUR-NS	CUR	BDP	BDP-NS
ED%	57.0 ± 0.9	81.9 ± 1.1	83.4 ± 3.7	65.5 ± 4.9
FPD (mg)	7.8 ± 0.3	6.8 ± 0.8	6.1 ± 0.1	7.6 ± 0.2
FPF (%)	60.3 ± 1.9 ^§^	64.7 ± 4.0 ^§^	62.7 ± 0.5 ^§^	68.1 ± 7.2 ^§^
MMAD (µm)	4.1 ± 0.1	3.4 ± 0.6	3.8 ± 0.1	3.7 ± 0.2
GSD	2.6 ± 0.1	3.1 ± 0.4	2.9 ± 0.1	2.6 ± 0.1

## Data Availability

Not applicable.

## Supplementary Materials

The following are available online at https://www.mdpi.com/article/10.3390/pharmaceutics13081300/s1, Figure S1: DSC curve of pure CUR, Figure S2: DSC curve of pure P188, Figure S3: DSC curve of the CUR-NS, Figure S4: DSC curve of pure BDP, Figure S5: DSC curve of the physical mixture of BDP/P188., Figure S6: DSC curve of the BDP-NS.
