# Supplementary material for: Pulmonary Delivery of Curcumin and Beclomethasone Dipropionate in a Multicomponent Nanosuspension for the Treatment of Bronchial Asthma
Source: Pharmaceutics. 2021 Aug 20;13(8):1300. doi: 10.3390/pharmaceutics13081300 (PMC8401312; doi:10.3390/pharmaceutics13081300)
Supplement: Supplementary file 1 [file pharmaceutics-13-01300-s001.zip › pharmaceutics-1328816-supplementary.pdf]

pharmaceutics

MDPI

Supplementary Materials: Pulmonary Delivery of Curcumin and Beclomethasone Dipropionate in a Multicomponent Nanosuspension for the Treatment of Bronchial Asthma

Luca Casula, Francesco Lai, Elena Pini, Donatella Valenti, Chiara Sinico, Maria Cristina Cardia, Salvatore Marceddu, Giorgia Ailuno and Anna Maria Fadda

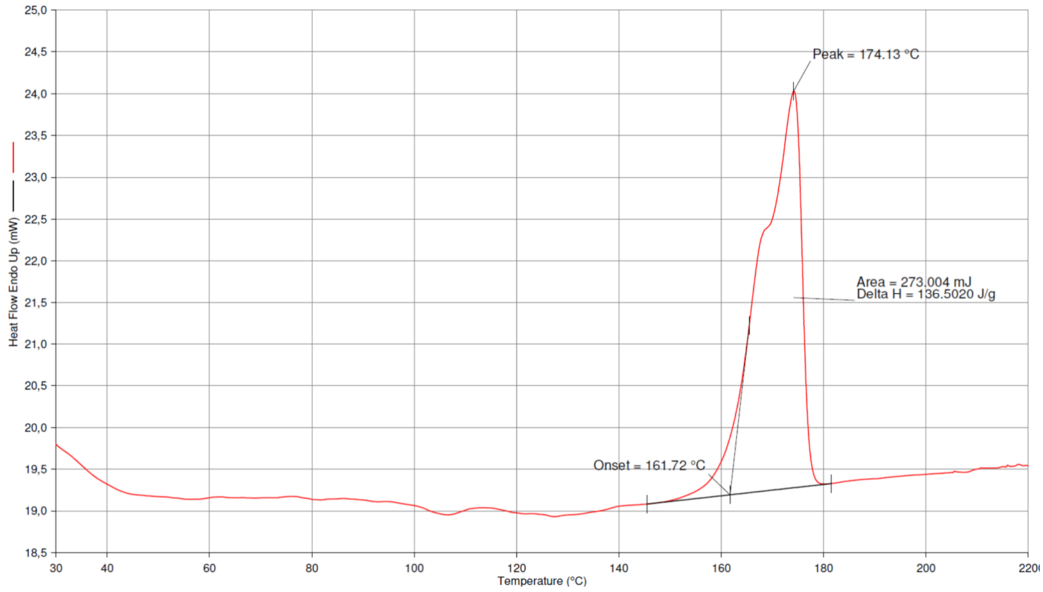

Figure S1. DSC curve of pure CUR.

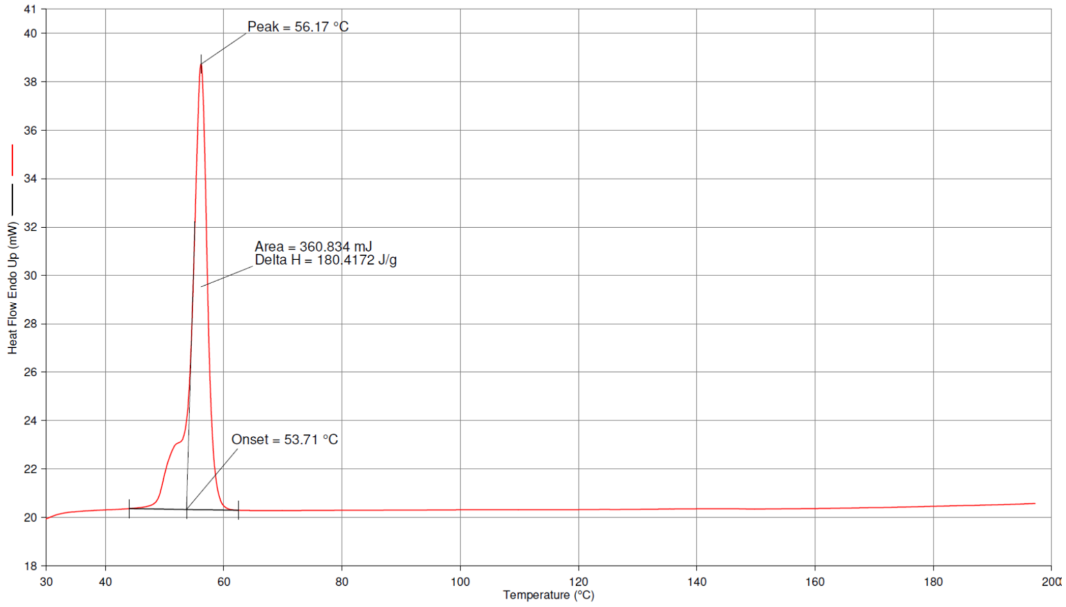

Figure S2. DSC curve of pure P188.

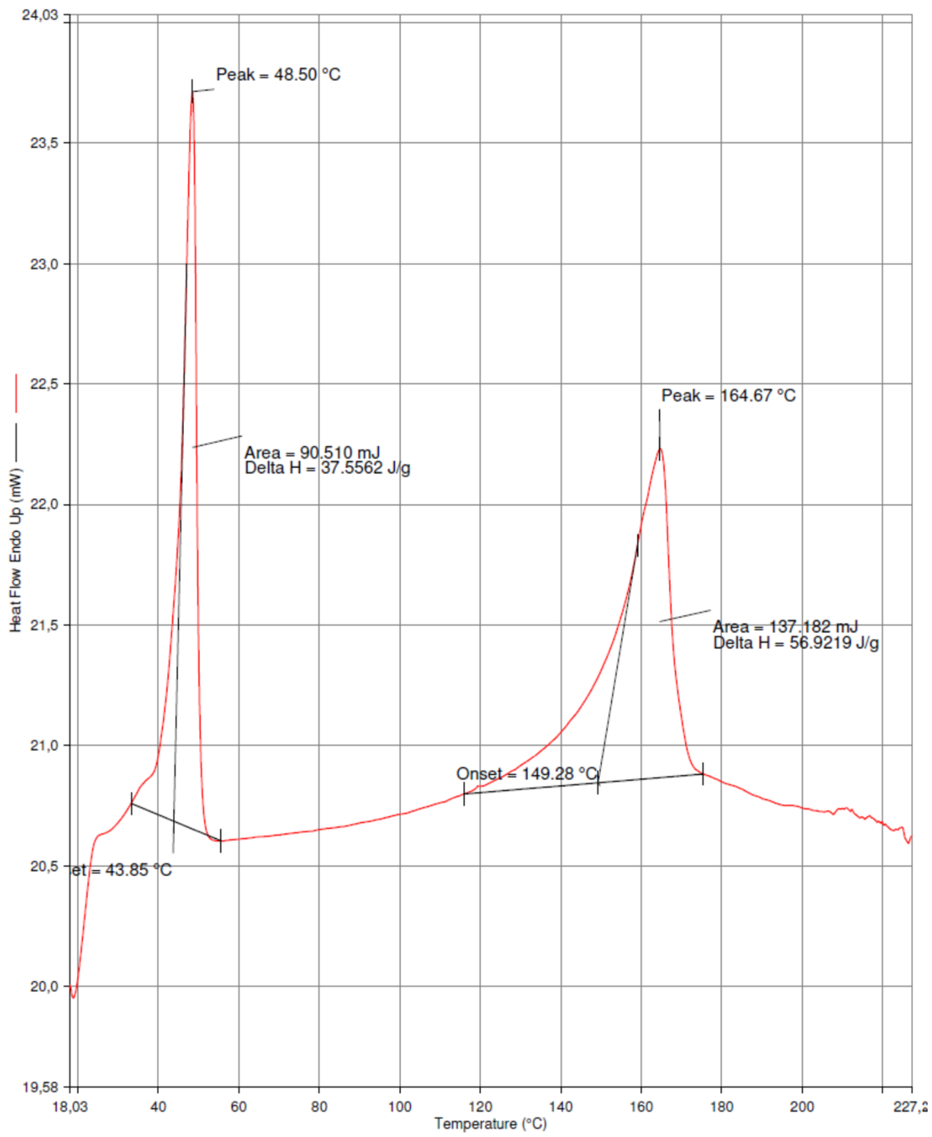

Figure S3. DSC curve of the CUR-NS.

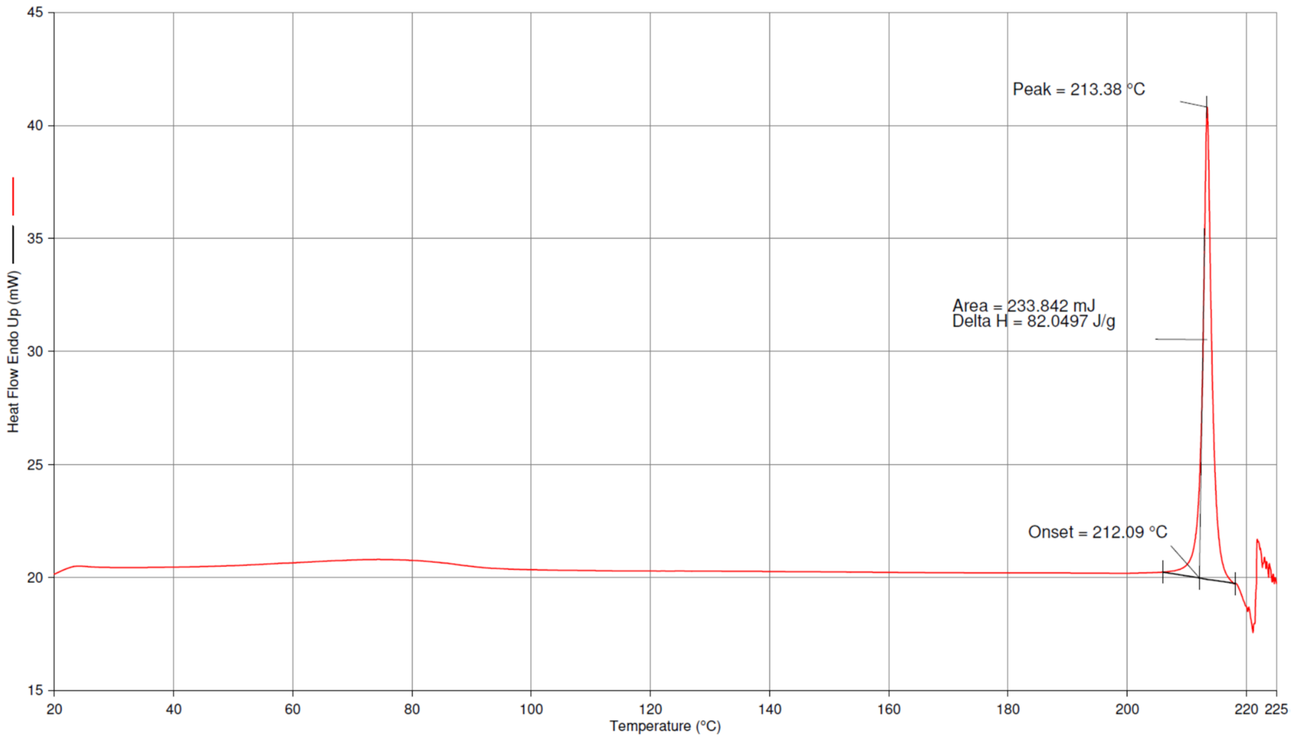

Figure S4. DSC curve of pure BDP.

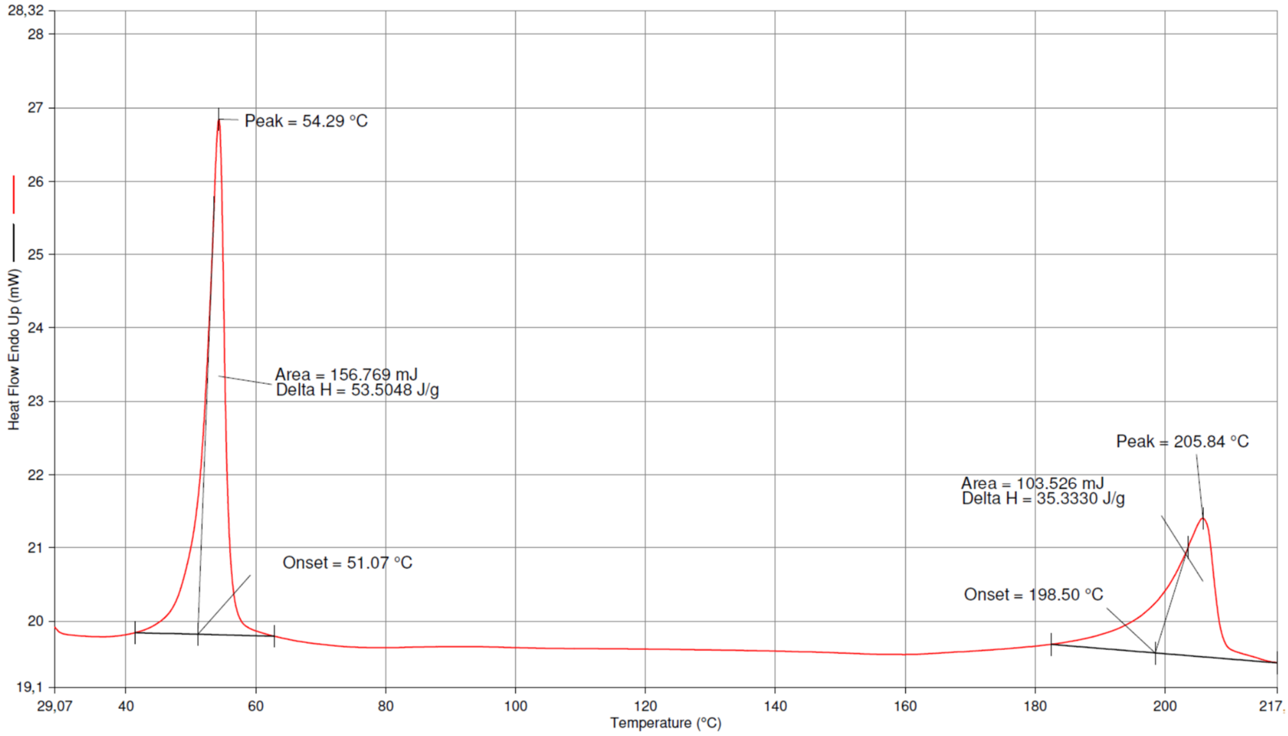

Figure S5. DSC curve of the physical mixture of BDP/P188.

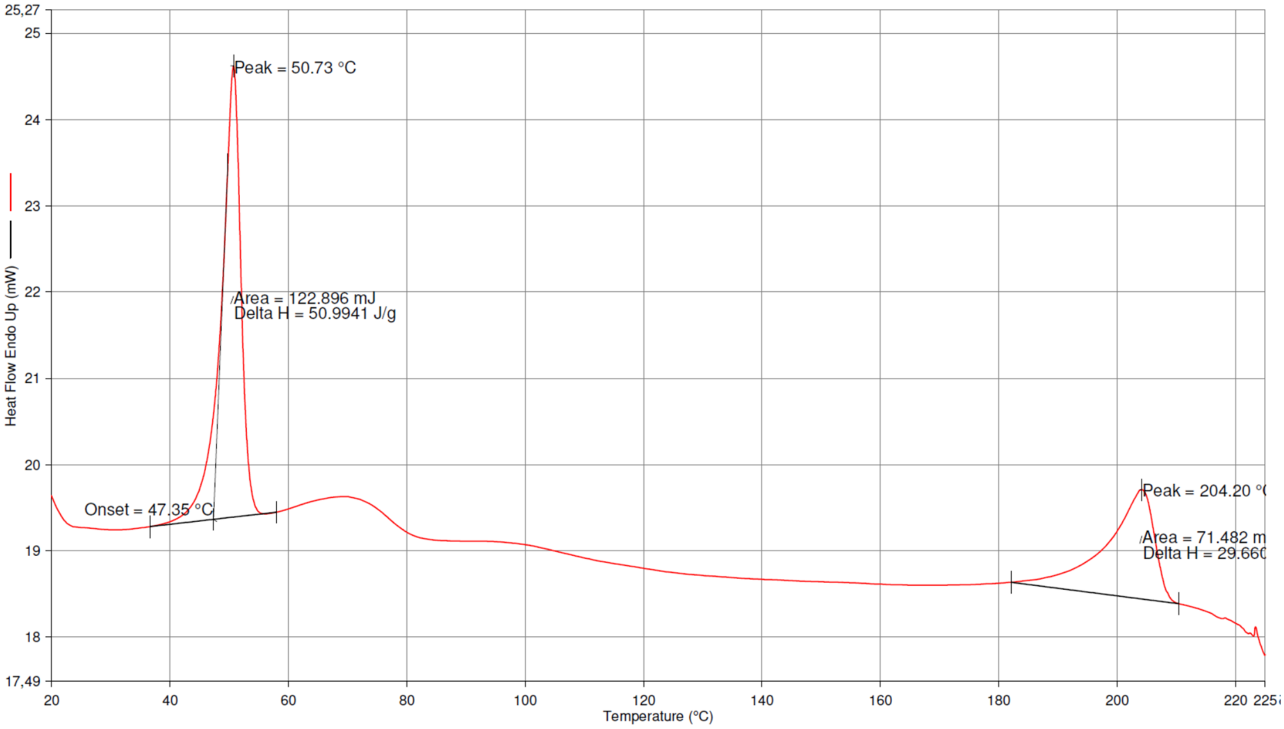

Figure S6. DSC curve of the BDP-NS.
